# Blood DNA methylation and breast cancer risk: a prospective nested case–control study

**DOI:** 10.1016/j.ebiom.2026.106352

**Published:** 2026-06-25

**Authors:** Juan Rodriguez, Felix Grassmann, Damien Kaukonen, Sara Hägg, Keith Humphreys, Mikael Eriksson, Yuqi Zhang, Katharina Morawitz, Marike Gabrielson, Per Hall, Kamila Czene

**Affiliations:** a Department of Medical Epidemiology and Biostatistics, Karolinska Institutet, Stockholm, Sweden; b Health and Medical University, Potsdam, Germany; c InFLAMES Research Flagship Center, University of Turku, Turku, Finland; d Department of Oncology, Södersjukhuset, Stockholm, Sweden

**Keywords:** Breast cancer, Blood-based biomarkers, DNA methylation, MRS, Risk prediction

## Abstract

**Background:**

Blood tests that predict breast cancer (BC) risk before diagnosis could complement mammography screening. Whole-blood DNA methylation is stable and may capture early systemic changes related to tumour development.

**Methods:**

We conducted a prospective matched case–control study nested within Sweden’s mammography screening programme, profiling whole-blood DNA methylation on the Illumina MethylationEPIC array in 377 female participants who later developed BC and 378 age-matched female controls. We identified methylation sites (CpG sites) in Stockholm and validated them in Skåne, after stringent quality control and adjustment for technical and epidemiological confounders. We then constructed a methylation risk score (MRS).

**Findings:**

We identified 87 CpG sites in Stockholm (FDR < 0.05; |β| > 0.015), of which 22 validated in Skåne (P < 0.05; concordant direction) and formed the MRS. The MRS demonstrated significant associations with BC risk, was consistent across different BC subtypes, and independent of established risk factors. The association of MRS with BC remained relatively stable over a pre-diagnostic window of up to five years. MRS exhibited predictive performance comparable to a polygenic risk score, and it significantly improved the 3–5-year prediagnosis discrimination of established BC risk models (P < 0.05, DeLong’s test). Several CpG sites correlated with immune-related genes, suggesting mechanisms involving immune modulation and tumour progression.

**Interpretation:**

Our methylation signature was significantly associated with BC risk and may complement existing risk models, particularly for estimating 3–5-year risk. These findings suggest that blood DNA methylation may support BC risk stratification, although further validation and assessment of clinical utility are needed.

**Funding:**

Swedish Research Council; Swedish Cancer Society; Stockholm County Council; Cancerföreningen i Stockholm; Erling-Persson Foundation; Sjöberg Foundation; and EU funds via ILB.

**Research in context**

**Evidence before this study**

Published prospective studies suggest that global methylation measures, epigenetic ageing metrics, and selected methylation markers in blood can be associated with later breast cancer, but many studies relied on a single prediagnostic blood sample, had limited external replication, and infrequently quantified incremental value beyond established clinical, genetic, or imaging-based risk tools. PubMed and Google Scholar were searched through December 2025 using combinations of “DNA methylation”, “blood-based”, “biomarkers”, and “breast cancer”.

**Added value of this study**

Within a population mammography screening programme, we discovered and validated a 22-marker blood DNA methylation signature in independent regional sets, using stringent quality control and careful adjustment for confounding and technical variation. We assessed how early the signal is informative using repeat samples from the same individual, benchmarked performance against established risk tools, and replicated the signal in an independent cohort profiled on a different methylation array platform.

**Implications of all the available evidence**

Blood DNA methylation signatures can provide short-to mid-term risk information and may help identify individuals who could benefit from tailored screening strategies. Before clinical adoption, larger studies should validate performance across diverse populations, simplify measurement to a targeted assay, and evaluate calibration, clinical utility, cost-effectiveness, and acceptability in real-world screening pathways.

## Introduction

Early detection of breast cancer (BC) is essential for improving patient outcomes.^1^ Mammographic screening has reduced BC mortality,^2^ but its effectiveness is limited by reduced sensitivity in dense breast tissue and challenges in detecting fast-growing tumours.^3^^,^^4^ Advances in genomic and epigenomic technologies now highlight blood-based biomarkers as minimally invasive tools that can enhance early detection.^5^ These biomarkers may offer predictive information years before clinical diagnosis.^6^ Among these, DNA methylation patterns have emerged as key regulatory mechanisms involved in breast carcinogenesis, and detectable in peripheral blood prior to symptom onset.^7^

Multiple recent studies have supported the clinical relevance of DNA methylation signatures for BC risk prediction, highlighting consistent associations of global DNA hypomethylation^8^ and accelerated epigenetic ageing with increased BC risk.^9^ However, further validation of blood-based methylation biomarkers is needed in large, population-based screening settings,^10^ especially through de novo epigenome-wide discovery approaches and within shorter pre-diagnostic windows more relevant to near-term BC risk stratification. Further, the integration of methylation markers with established BC clinical risk prediction models, such as polygenic risk score (PRS), and established risk algorithms (e.g., Tyrer-Cuzick^11^ and AI Risk Model^12^), requires rigorous evaluation to establish clinical utility and feasibility.^13^ Moreover, the standard approach in previous studies has been to analyse only a single pre-diagnostic sample, leaving the temporal patterns of methylation signals uncertain.

This study aims to address the key gaps in prior studies on blood-based DNA methylation biomarkers for BC risk prediction. Within the unique KARolinska MAmmography Project for Risk Prediction of Breast Cancer (KARMA) screening cohort from Sweden,^14^ we identified methylation sites associated with BC in matched case–control pairs, summarised this signal as a methylation risk score (MRS),^15^ and evaluate whether the MRS provides complementary discrimination beyond existing BC risk models. We also aimed to assess the temporal stability of the methylation signal using repeated pre-diagnostic blood samples from the same individual. Finally, we examined replication in an independent external cohort.

## Methods

### Study population

Individuals aged 40–74 years with a female-coded Swedish personal identity number are invited every 18–24 months to the Swedish National Mammography Screening Programme. Individuals attending screening in the Stockholm and Skåne regions were invited to the KARMA study between 2011 and 2013. 70,877 individuals consented to participate, answered a comprehensive web-based questionnaire, donated blood, and allowed linkage to national registers.^14^ Using linkage to the Swedish Cancer Registry, we identified all individuals within the KARMA cohort who received a first BC diagnosis between 2012 and 2015, occurring between 6 months and 3 years of blood draw. Controls were selected from KARMA participants without incident BC using MatchIt::matchit in R (v4.7.1)^16^ to achieve a similar median age at blood draw between cases and controls (median matching). Baseline characteristics for the combined cohort and for each regional set are presented in Supplementary Table S1. At blood draw, the mean (standard deviation, SD) age was 58.8 (9.1) years in cases and 58.8 (9.7) years in controls; mean (SD) BMI was 25.5 (4.3) and 25.3 (4.3) kg/m^2^, respectively. No randomisation or experimental intervention was performed. No formal a priori power calculation was performed. Sample identifiers did not encode case–control status. Laboratory personnel performing DNA extraction, bisulfite conversion, and array hybridisation were blinded to case–control status. Cases and controls were randomised on the arrays. Of note, repeated samples from the same participant were intentionally placed on the same plate to reduce between-plate variation in within-person comparisons.

Information on menopausal status, BMI, and smoking was obtained from questionnaires. Family history in first-degree relatives was retrieved from the Swedish Multi-generation Registry.^17^ Tumour characteristics such as oestrogen receptor (ER) status, human epidermal growth factor receptor 2 (HER2) status, grade and lymph node involvement were retrieved from medical records or from the Swedish Quality Register for Breast Cancer. Mode of detection (interval or screen-detected BC) was defined by the timing between the last mammographic screening and time of diagnosis, as previously described.^18^

### Sample collection and timing

The final study cohort comprised female participants enrolled in screening programmes in Stockholm (discovery set; 196 BC cases and 191 age-matched controls) and Skåne (validation set; 181 BC cases and 187 age-matched controls). All BC cases in both sets provided blood samples between 6 months and 3 years prior to their BC diagnosis (median = 2.07 years), with the vast majority (≈92%) obtained between 1 and 2.5 years before diagnosis. These samples are referred to as index blood draws, collected at the last negative screening visit preceding diagnosis. This timing aligned with our primary objective of developing an MRS that predicts BC within ∼2 years of sampling. Additionally, for a subset of cases, a secondary blood draw was available either before (N = 96 cases; 3–5 years before BC diagnosis) or after (N = 59 cases; <6 months before diagnosis) the index mammography visit, enabling longitudinal analysis of methylation profiles. The distribution of time from blood draw to diagnosis is illustrated in Supplementary Fig. S1A (and separately for Stockholm and Skåne in Supplementary Fig. S1B and C).

### DNA methylation analysis

Genomic DNA was isolated from peripheral whole-blood samples. For each participant, ≥1 μg DNA in ≥20 μL per sample was submitted to the SNP&SEQ Technology Platform (Uppsala University, Sweden), where concentrations were verified using Quant-iT PicoGreen dsDNA assay. DNA was bisulfite-converted using the EZ DNA Methylation Kit (Zymo Research, Orange, CA, USA; Product No: D5004) according to the manufacturer’s protocol. Bisulfite-converted DNA was then hybridised to the Infinium MethylationEPIC BeadChip v1.0 B5 (Illumina Inc., San Diego, CA, USA). Initial array quality control (QC) was performed in Illumina GenomeStudio 2011.1 (Illumina Inc., San Diego, CA, USA; RRID:SCR_010973). Raw IDAT files were exported for downstream analysis. The obtained methylation data then went through a rigorous multistep QC pipeline: First, low-quality probes (detection P > 0.05 or > 10% missing values), probes overlapping SNP sites, and probes on sex chromosomes were removed, leaving 631,159 CpGs. Second, background correction and normalisation were applied, followed by cell-type adjustment (using residuals from linear regression of each CpG on DNA methylation–based estimates of eight blood cell types) and batch effect correction. Finally, probes significantly correlated with BeadChip row (P < 0.05 and | β | >0.005 per % methylation across any row-level comparison) were excluded, resulting in a final analysis dataset of 429,953 CpGs. This last filter was included because several studies have demonstrated systematic “row effects” in median methylation intensities across the eight physical rows of the Infinium BeadChip.^19^, ^20^, ^21^, ^22^ To ensure these artefacts did not drive our results, we applied a regression-based test to an independent cohort of 375 Swedish population controls^23^ processed in parallel. A detailed flowchart of the QC pipeline can be found in Supplementary Fig. S2. Processing was performed in R (v4.4.3), with background correction using methylumi::noob (v2.52.0),^24^ and normalisation with watermelon::dasen (v2.12.0).^25^ Cell-type composition was estimated using the Houseman algorithm (minfi::estimateCellCounts; v1.52.1).^26^ Batch effects (determined from the array barcodes) were corrected with sva::ComBat (v3.54.0).^27^ Principal component (PC) analysis of QC-passed and normalised CpGs is shown in Supplementary Fig. S3.

### MRS generation and BC risk models retrieval

To generate an MRS, we multiplied individual CpG methylation levels by their corresponding β coefficients. The resulting weighted CpG values were summed for each participant and scaled (z-score transformation). For the primary analyses, β coefficients from the pooled Stockholm and Skåne sets were used as weights to construct an MRS. For predictive performance in the training/test split framework, βs were estimated exclusively from the training set (Stockholm) and applied to Skåne.

We compared our MRS with three established risk tools. First, we used a Karolinska-developed image-based AI risk model,^12^^,^^28^ that has shown strong performance identifying individuals at short-term high risk (∼0–2 years) of BC, and validated across multiple European screening programmes.^29^ Second, we obtained 5-year absolute risk from the Tyrer-Cuzick (IBIS v8) algorithm, which integrates clinical and family-history factors with mammographic density.^11^ Third, we used the 313-SNP PRS,^30^^,^^31^ which is the allele-dosage-weighted sum of 313 common risk variants, with higher scores indicating greater common-variant BC risk. For more information regarding PRS computation, see ref.^32^

### Statistical analysis

Analyses were conducted in R (v4.4.3). Epigenome-wide association analyses were run separately within the Stockholm discovery set and the Skåne validation set. At each of the 429,953 CpG sites, we fitted linear regression models of methylation level on case–control status with adjustment for age at blood draw, BMI (quartile-based assessed at the time of study entry, with missing coded as a separate category), smoking status (never, former, current, missing), and the first three methylation-derived PCs to account for residual technical variation. The combined regression analysis of Stockholm and Skåne additionally included recruitment centre as a covariate.

We used logistic regression to assess associations of the 22 significant CpG sites and the MRS with tumour characteristics. For CpGs, results were displayed as a correlation heatmap, generated with corrplot::corrplot (v0.95).^33^ For the MRS, analyses were restricted to BC cases and shown as forest plots of odds ratios (ORs) per SD increase with 95% confidence intervals (CIs), using metafor::forest (v4.8.0).^34^ The results of the association of MRS with BC risk over time were plotted as violin plots with ggplot2::ggplot (v3.5.2).^35^ Discriminative performance was evaluated using Area Under the Curve (AUC) estimates from receiver operating characteristic (ROC) analysis, and statistical significance of AUC improvements was assessed using DeLong’s test via pROC::roc.test (v1.18.5).^36^

### Ethics approval and consent to participate

All individuals gave written informed consent to participate in the study, to the retrieval of information from medical records, national registries and mammographic images; donated blood at enrolment for genetic analysis and answered a detailed questionnaire about background and lifestyle risk factors. The study was conducted in accordance with the Declaration of Helsinki. The study was approved by the Regional Ethical Review Board in Stockholm, Sweden (Dnr 2010/958-31/1, 2012/217/-32/2 and 2014/1401-32).

### Role of the funder

The funders had no role in the design and conduct of the study; collection, management, analysis, and interpretation of the data; preparation, review, or approval of the manuscript; or the decision to submit the manuscript for publication. No author was paid by a pharmaceutical company or other agency to write this article. Authors were not precluded from accessing data in the study and accept responsibility for the decision to submit for publication.

## Results

### Genome-wide DNA methylation analysis

The final Swedish study cohort included 377 BC cases and 378 controls, comprising a Stockholm discovery set (196 cases, 191 controls) and an independent Skåne validation set (181 cases, 187 controls). All cases contributed an index blood sample collected 6 months to 3 years before diagnosis (median 2.07 years), and repeated blood samples were available for subsets of cases 3–5 years before diagnosis (N = 96) or <6 months before diagnosis (N = 59). We identified 87 CpG sites significantly associated with BC risk that passed an FDR threshold (<0.05) and a minimum effect-size criterion (|β| > 0.015 between cases and controls) in the discovery set (Stockholm). The |β| > 0.015 threshold was selected as a pragmatic intermediate effect-size filter, informed by EWAS recommendations, to balance exclusion of very small differences potentially attributable to technical variation with retention of modest pre-diagnostic blood-based methylation signals.^37^ Among these 87 CpG sites, 22 were successfully validated in the independent Skåne set (P < 0.05, concordant direction). Of note, 2 CpG sites (cg10769767 and cg17100154) reached statistical significance in both sets but showed opposite directions of associations and were therefore not considered validated. Among the 22 validated CpGs, 18 (81.8%) showed decreased methylation in cases relative to controls, and 4 (18.2%) showed increased methylation. Summary statistics for validated CpG sites, and per-CpG associations with BC across sets and in the pooled meta-analysis, are presented in Table 1, together with the corresponding ORs for the pooled meta-analysis. A Manhattan plot of epigenome-wide association P values across chromosomes is shown in Supplementary Fig. S4. The intercorrelation of the 22 CpG sites was assessed using pairwise Pearson correlations of methylation β-values across all participants (Supplementary Fig. S5). Correlations were modest (all |r| ≤ 0.60), indicating limited redundancy across sites. Variance inflation factors (VIFs) from a multivariable model including all 22 validated CpGs were also modest, with a maximum VIF of 2.09 (range 1.10–2.09), indicating no evidence of severe collinearity.

**Table 1:** Summary statistics for the 22 CpG sites significantly associated with breast cancer in Stockholm and validated in Skåne.

CpG information			Set									Pooled meta-analysis OR (CI 95%) per SD		
			Stockholm			Skåne			Pooled meta-analysis					
CpG ID	Median β Cases	Median β Controls	Δβ	SE	P	Δβ	SE	P	Δβ	SE	P	Raw -Unadjusted	Direction-harmonised	+ Adj. for Age, Centre, BMI, Smoking
cg23621438	0.721	0.732	−0.017	0.002	3.25E-14	−0.010	0.002	7.68E-07	−0.014	0.001	5.5E-19	0.53 (0.45–0.62)	1.88 (1.6–2.22)	1.97 (1.67–2.34)
cg00549040	0.559	0.569	−0.018	0.003	6.89E-11	−0.011	0.002	1.60E-05	−0.014	0.002	1.67E-14	0.57 (0.48–0.66)	1.76 (1.51–2.07)	1.84 (1.57–2.17)
cg23826579	0.702	0.719	−0.016	0.003	5.39E-08	−0.013	0.003	3.45E-06	−0.014	0.002	5.59E-12	0.6 (0.51–0.69)	1.68 (1.44–1.96)	1.71 (1.46–2.01)
cg15559737	0.747	0.758	−0.016	0.003	1.06E-09	−0.009	0.003	5.10E-04	−0.012	0.002	1.13E-11	0.6 (0.51–0.7)	1.67 (1.43–1.95)	1.7 (1.46–2.01)
cg16528891	0.689	0.7045	−0.017	0.003	3.02E-07	−0.013	0.003	5.91E-05	−0.014	0.002	4.19E-10	0.62 (0.53–0.72)	1.62 (1.39–1.89)	1.63 (1.39–1.91)
cg27408262	0.767	0.7805	−0.017	0.003	1.29E-08	−0.008	0.003	4.40E-03	−0.013	0.002	5.77E-10	0.62 (0.53–0.72)	1.6 (1.38–1.87)	1.61 (1.38–1.88)
cg24375690	0.566	0.584	−0.023	0.003	2.77E-10	−0.008	0.004	2.52E-02	−0.015	0.003	2.71E-09	0.63 (0.54–0.74)	1.58 (1.36–1.84)	1.59 (1.36–1.87)
cg03770187	0.645	0.659	−0.019	0.003	2.21E-08	−0.010	0.004	7.31E-03	−0.014	0.002	8.94E-09	0.65 (0.56–0.76)	1.53 (1.32–1.78)	1.54 (1.32–1.8)
cg16984151	0.732	0.7475	−0.020	0.004	9.34E-08	−0.010	0.004	1.27E-02	−0.015	0.003	1.91E-08	0.64 (0.55–0.75)	1.56 (1.34–1.82)	1.55 (1.33–1.83)
cg04252957	0.815	0.827	−0.016	0.003	1.57E-08	−0.007	0.003	1.72E-02	−0.012	0.002	2.46E-08	0.65 (0.55–0.75)	1.55 (1.33–1.81)	1.55 (1.33–1.82)
cg12379755	0.656	0.667	−0.016	0.003	4.61E-07	−0.010	0.003	1.71E-03	−0.013	0.002	2.77E-08	0.65 (0.55–0.75)	1.55 (1.33–1.81)	1.56 (1.34–1.84)
cg13417679	0.586	0.605	−0.017	0.004	7.44E-05	−0.016	0.004	1.32E-04	−0.016	0.003	4.46E-08	0.69 (0.6–0.8)	1.44 (1.25–1.68)	1.46 (1.26–1.71)
cg21240283	0.472	0.486	−0.016	0.004	2.11E-05	−0.013	0.004	1.03E-03	−0.015	0.003	1.08E-07	0.67 (0.58–0.78)	1.49 (1.28–1.73)	1.5 (1.28–1.75)
cg00321115	0.756	0.764	−0.016	0.003	2.08E-07	−0.008	0.003	1.97E-02	−0.012	0.002	1.82E-07	0.68 (0.59–0.79)	1.47 (1.26–1.71)	1.49 (1.28–1.74)
cg15975895	0.572	0.558	0.017	0.004	8.6E-05	0.016	0.004	3.17E-04	0.016	0.003	1.83E-07	1.4 (1.21–1.62)	1.4 (1.21–1.62)	1.45 (1.25–1.7)
cg17201343	0.464	0.447	0.016	0.004	5.72E-05	0.014	0.004	4.64E-04	0.015	0.003	2.43E-07	1.42 (1.23–1.65)	1.42 (1.23–1.65)	1.44 (1.23–1.68)
cg15947658	0.645	0.66	−0.020	0.003	1.72E-08	−0.008	0.004	4.86E-02	−0.013	0.003	3.77E-07	0.67 (0.58–0.78)	1.49 (1.28–1.73)	1.48 (1.27–1.72)
cg10701847	0.727	0.738	−0.016	0.003	3.29E-06	−0.007	0.003	3.07E-02	−0.011	0.002	1.22E-06	0.69 (0.59–0.8)	1.46 (1.26–1.7)	1.45 (1.25–1.7)
cg24790837	0.648	0.663	−0.016	0.003	7.67E-06	−0.008	0.003	2.81E-02	−0.012	0.002	1.29E-06	0.69 (0.6–0.8)	1.44 (1.25–1.68)	1.46 (1.25–1.7)
cg16412995	0.762	0.751	0.015	0.004	7.46E-05	0.009	0.003	1.26E-02	0.012	0.003	3.71E-06	1.4 (1.2–1.62)	1.4 (1.2–1.62)	1.42 (1.22–1.66)
cg26008533	0.653	0.6675	−0.020	0.004	6.99E-06	−0.009	0.004	3.70E-02	−0.014	0.003	6.52E-06	0.75 (0.64–0.86)	1.34 (1.16–1.55)	1.36 (1.17–1.58)
cg03313542	0.435	0.4095	0.028	0.007	3.79E-05	0.015	0.007	2.37E-02	0.021	0.005	9.25E-06	1.4 (1.21–1.62)	1.4 (1.21–1.62)	1.41 (1.21–1.65)
**Methylation risk score (MRS)**													**2.72 (2.25–3.30)**	**2.92 (2.39–3.57)**

Columns list each CpG identifier and the median methylation β-value in all index breast cancer cases and controls. For all CpGs, methylation β-values were available for all index breast cancer cases (N = 377) and controls (N = 378). Association statistics are shown separately for Stockholm (discovery set), Skåne (validation set), and the pooled meta-analysis. The final columns report ORs and 95% CI for BC risk per 1-SD increase in β-value: (i) unadjusted; (ii) direction-harmonised (expressed so that OR > 1 indicates higher risk with higher methylation); and (iii) direction-harmonised adjusted for age, recruitment centre, BMI, and smoking. The blue-highlighted final row shows the methylation risk score (MRS), computed as a weighted sum of the listed CpGs, with its corresponding ORs.

CI, confidence interval; OR, odds ratio; SD, standard deviation.

Next, to investigate the joint contribution of the 22-BC-associated CpG sites, we computed an MRS. The last row in Table 1 shows the association of the computed MRS with BC risk (Overall MRS), based on the aggregate values of these validated CpGs after being multiplied by their effect sizes. Among index blood draw samples (6 months to 3 years prior to diagnosis), each SD increase in MRS was associated with a significantly increased BC risk: OR, 2.72 (95% CI, 2.25–3.30). Adjusting for the main confounders did not alter the observed association: OR, 2.92 (95% CI 2.39–3.57). The MRS was minimally influenced by major BC risk factors, such as age, family history of BC, lifestyle factors, and established BC risk prediction models, thus indicating that it provides independent predictive information (Supplementary Fig. S6).

### Association of identified CpG sites and MRS with tumour characteristics

**Fig. 1:**
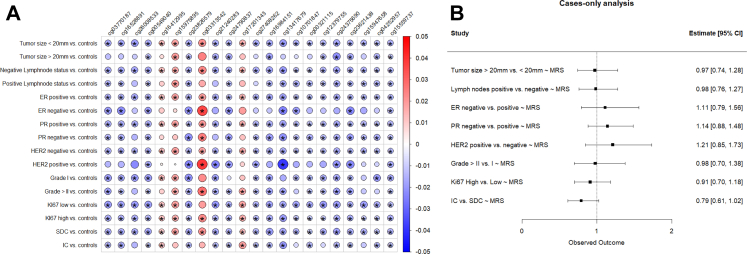
**Association of identified CpGs and MRS with tumour characteristics. Panel A** shows the association of validated CpGs with tumour characteristics among index blood draws cases and controls using a dot plot. Each dot represents the association, with colour indicating the direction and strength of the association (blue for negative, red for positive). The size of the dots reflects the significance of the association. The asterisks show a significant association (Bonferroni-corrected P < 0.0023 (0.05/22)). Models were adjusted for age at blood draw, BMI, smoking status, and recruitment centre. **Panel B** is a forest plot showing ORs and 95% CIs for the association between the MRS and tumour characteristics among BC cases only, adjusting for age at diagnosis, BMI, smoking status, and recruitment centre. For both panels, tumour characteristics included ER, PR, and HER2 receptor status, grade, Ki67 proliferation index, lymph node involvement, tumour size, and detection mode (IC vs. SDC). IC was defined as cancer diagnosed after a negative screen and before the next scheduled screening round; SDC as cancer diagnosed at a routine screening visit. Females lacking attendance at the scheduled pre-diagnosis screening were excluded from IC-SDC analyses. Ki-67 was categorised as low (<10%) and high (≥20%); values between 10% and <20% were considered intermediate. The vertical dashed line indicates the null value (OR = 1). Abbreviations: *BC,* breast cancer; *CI,* confidence interval; *ER,* oestrogen receptor; *HER2,* human epidermal growth factor receptor 2; *IC,* interval breast cancer; *MRS,* methylation risk score; *OR,* odds ratio; *PR,* progesterone receptor; *SDC,* screen-detected breast cancer.

Fig. 1 summarises associations of individual CpGs and the aggregate MRS with tumour features. At the individual-CpG level (panel A; case–control analysis), multiple validated CpG sites were associated with BC after Bonferroni correction (P < 0.0023). Associations were observed across clinical strata, although fewer sites were Bonferroni-significant in the ER-negative, HER2-positive, and tumour size >20 mm strata, likely due to lower sample size. At the aggregate-score level (panel B; case-only analysis), a 1-SD increase in the MRS showed no clear association with aggressiveness. Some point estimates differed by subtype (e.g., ER status and detection mode), but differences were small and not considered hypothesis-generating.

### Association of MRS with BC risk over time

**Fig. 2:**
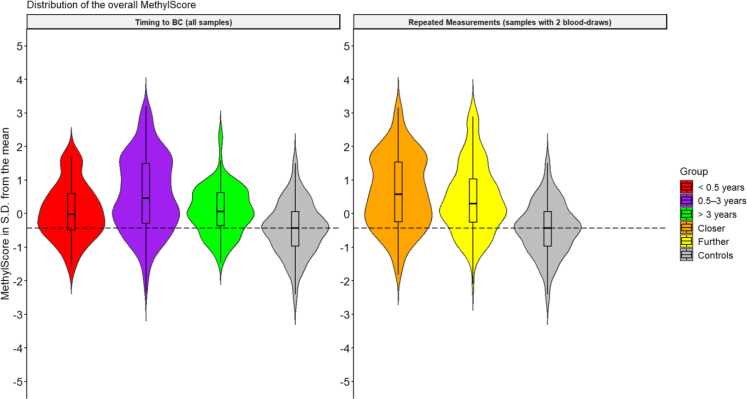
**Distribution of MRS in patients with breast cancer vs. controls over time.** The figure presents violin plots of MRS across patients with BC and matched controls over time. Each violin plot illustrates the distribution of MRS values (expressed as standard deviations from the mean), with an overlaid boxplot indicating the interquartile range and median. **LeftPanel**: All BC samples are stratified by time to diagnosis from the baseline blood draw into three intervals: <0.5 years, 0.5–3 years, and >3 years. **RightPanel**: A subset of patients with BC with two blood samples are categorised based on the proximity of the blood draw to diagnosis (closer vs. further) and compared to controls. Abbreviations: *BC,* breast cancer; *MRS,* methylation risk score.

The distribution of the MRS stratified by timing to diagnosis and repeated measurements is illustrated in Fig. 2. Patients exhibited higher MRS values compared with controls, and the values remained stable among cases during the study window (up to 5 years before diagnosis). Associations between MRS and BC risk by timing of blood draw are shown in Table 2. Across all time strata, individuals in the highest MRS tertile had significantly greater BC risk compared with those in the lowest tertile (e.g., OR per SD, 5.68 (95% CI 3.75–8.61) for index blood draws). Further adjusting for multiple established BC risk factors did not significantly alter the outcomes observed. Similar significant associations were observed for secondary blood draws collected for a subset of those patients, either closer to diagnosis (<6 months) or further away (3–5 years), suggesting the robustness of MRS as a predictive biomarker across different pre-diagnostic intervals.

**Table 2:** Meta-analysis of the association of MRS with breast cancer risk.

Association of MRS with BC risk MRS meta-analysis (Stockholm + Skåne)	No. Cases	No. Controls	Adj. for Age + Centre + BMI + Smoking OR (95% CI) per SD	+ Breast density + Parity + Age at first birth + Menarche age + Education level + Alcohol intake + Family history BC + Benign breast disorder OR (95% CI) per SD
Index blood draw 0.5–3-year to BC diagnosis	377	378	**2.92 (2.39–3.57)**	**2.93 (2.39–3.57)**
MRS tertiles				
0%–33.3%	48	126	Referent	Referent
33.3%–66.6%	77	126	**1.68 (1.07–2.63)**	**1.61 (1.01–2.56)**
66.6%–100%	252	126	**5.68 (3.75–8.61)**	**5.55 (3.61–8.53)**
Secondary blood-draw 3–5-year to BC diagnosis	96	378	**2.07 (1.59–2.69)**	**2.15 (1.62–2.86)**
MRS tertiles				
0%–33.3%	12	126	Referent	Referent
33.3%–66.6%	22	126	1.86 (0.87–3.99)	2.01 (0.91–4.42)
66.6%–100%	62	126	**4.97 (2.51–9.82)**	**5.42 (2.65–11.11)**
Secondary blood-draw < 0.5 years to BC diagnosis	59	378	**2.08 (1.52–2.84)**	**2.29 (1.61–3.27)**
MRS tertiles				
0%–33.3%	8	126	Referent	Referent
33.3%–66.6%	17	126	**2.59 (1.02–6.59)**	**3.13 (1.16–8.46)**
66.6%–100%	34	126	**5.61 (2.34–13.48)**	**6.49 (2.51–16.80)**

Association of MRS with BC risk, stratified by time to diagnosis in Sweden. MRS was further stratified by tertiles for each subgroup (based on controls). Index samples (in purple) represent those samples with a blood-draw taking during the last negative screening. Secondary blood-draws are divided into those with a blood-draw before the last negative mammographic screening (green, between 3 and 5 years to BC diagnosis), and those with a blood-draw with less than half a year to BC diagnosis (red). Additional BC risk factors were accounted for in the model on the last column to the right. Age at first birth and menarche age variables are combined into a single reproductive-history variable, with categories for nulliparous females, parous females with first birth before age 30, parous females with first birth at age 30 or later, and females with unknown parity. NOTE: Significant associations (P < 0.05) are denoted in bold.

BC, breast cancer; CI, confidence interval; MRS, methylation risk score; OR, odds ratio.

### Predictive performance of MRS by time

Table 3 shows the overall discrimination and incremental gain of the MRS relative to established BC risk models (the 2-year AI Risk Model, 5-year Tyrer-Cuzick [IBIS v8], and a 313-SNP PRS). MRS performance was evaluated in two ways: first in the combined sets (development and testing within the pooled Stockholm + Skåne samples) and second using a training/test split approach (training in Stockholm and testing in Skåne, data not shown in tables). MRS discrimination was consistent across both approaches and time intervals, yielding AUCs slightly higher than those of the 313-SNP PRS and the Tyrer-Cuzick model under the most conservative specification.

**Table 3:** Performance of breast cancer risk-assessment models in Sweden.

Training/Test split (modelled in Stockholm; predicting in Skåne)					
Index blood draw 0.5–3 years to BC diagnosis	N cases	N controls	AUC (95% CI)	AUC adding external MRS (95% CI)	P
MRS	181	187	0.66 (0.61–0.72)	–	–
2-year AI Risk Model	157	187	0.74 (0.69–0.79)	0.76 (0.70–0.81)	0.7
5-year Tyrer-Cuzick	155	181	0.65 (0.59–0.71)	0.67 (0.61–0.73)	0.7
313-SNP PRS	157	124	0.62 (0.56–0.69)	0.69 (0.63–0.75)	0.054
Secondary blood draw *3–5 years to BC diagnosis*	N cases	N controls	AUC (95% CI)	AUC adding external MRS (95% CI)	P

MRS	47	187	0.71 (0.62–0.79)	–	–
2-year AI Risk Model	47	187	0.59 (0.51–0.69)	**0.71 (0.62–0.79)**	**0.011**
5-year Tyrer-Cuzick	46	181	0.58 (0.48–0.68)	**0.70 (0.62–0.79)**	**0.049**
313-SNP PRS	37	124	0.59 (0.48–0.69)	**0.74 (0.64–0.83)**	**0.003**

Discriminatory performance of the MRS, 2-year AI-based Risk model, 5-year Tyrer-Cuzick Risk Score, and 313-SNP PRS, stratified by time from blood draw to diagnosis and evaluated under a Training/Test split: the MRS was developed in the Stockholm set and evaluated in the Skåne set. The last column indicates whether adding the MRS improved discrimination relative to each model (DeLong’s test). NOTE: significant improvements (P < 0.05) are denoted in bold.

AUC, area under the curve; BC, breast cancer; CI, confidence interval; PRS, polygenic risk score; MRS, methylation risk score.

While the addition of the MRS in the combined sets approach produced statistically significant AUC gains relative to all comparator models, these results may be influenced by overfitting. We therefore emphasise the training/test split findings, which show no significant improvement for the 0.5–3-year pre-diagnosis discrimination, but significant gains for the 3–5-year pre-diagnosis window across all established models—AI (ΔAUC = 0.12; 0.59 → 0.71; *P* = 0.011), Tyrer–Cuzick v8 (ΔAUC = 0.12; 0.58 → 0.70; *P* = 0.049), and 313-SNP PRS (ΔAUC = 0.15; 0.59 → 0.74; *P* = 0.003). In an additional analysis, MRS performance showed no meaningful differences across mammographic density strata (AUC, 0.72 (95% CI 0.67–0.77) for PD > 25 vs. AUC, 0.73 (95% CI 0.69–0.77) for PD < 25; data not shown in tables). This suggests that it captures risk information that is not strongly driven by mammographic density and may therefore complement image-based risk models rather than overlap with them.

To independently validate our findings, we performed an external replication using pre-diagnostic whole-blood methylation data from EPIC-Italy (GSE51032), analysed using Illumina 450 K array. EPIC-Italy is a large prospective cohort assembled across multiple Italian centres, with blood collected at recruitment together with anthropometric, dietary, and lifestyle data, making it a particularly informative external replication setting because it differs from the Swedish screening cohorts in geography, background population, and array platform. In addition, EPIC-Italy spans a much broader pre-diagnostic window, with blood samples collected up to 17 years before BC diagnosis. After excluding male participants, the EPIC-Italy set comprised 228 cases and 340 controls. Raw intensities were processed and normalised using the same pipeline as in the Swedish analyses. Of the 22 CpGs in our signature, 13 were present on the 450 K array; 10/13 replicated with concordant direction (all P < 0.001), whereas 3/13 did not (Supplementary Fig. S7A). Using the 13 available CpGs and the pooled meta-analysis weights, the resulting MRS was associated with incident BC (OR per SD, 1.62; P < 0.00001) and achieved an AUC of 0.63 when all EPIC-Italy samples were combined, with cases having blood drawn as early as 17 years before BC diagnosis (data not shown in tables). The distribution of the resulting MRS on all EPIC-Italy samples stratified by timing to diagnosis is illustrated in Supplementary Fig. S7B. Notably, Supplementary Fig. S7C shows that the time-stratified AUCs were comparable to those observed in our study for 0.5–3 years (0.71, 95% CI 0.64–0.78) and 3–5 years (0.70, 95% CI 0.60–0.80), with performance near chance at 5–17 years (0.53, 95% CI 0.47–0.59).

### Functional annotation of validated CpGs

We annotated the 22 validated CpG sites to nearby genes and genomic features, using Infinium MethylationEPIC v1.0 B5 Manifest File^38^ (Supplementary Table S2). Several CpGs with decreased methylation in cases were located near immune-relevant genes —*MICA*, *FAM3C*, and *FLT1*/*VEGFR1*—with evidence of detectable expression in peripheral blood mononuclear cells.^39^, ^40^, ^41^ Other validated CpGs were found near genes involved in transcriptional regulation (*CTBP2*,^42^ *ZNF438*^43^), cholesterol biosynthesis (*DHCR7*^44^), zinc transport (*SLC30A6*^45^), and cell-cycle regulation (*CCNY*^46^).

## Discussion

We identified a 22-CpG MRS associated with future BC risk up to 5 years before diagnosis. Its associations were consistent across tumour subgroups, indicating that these methylation markers capture fundamental epigenetic changes in breast carcinogenesis. The discriminatory performance of the MRS was comparable to that of the 313-SNP PRS and the Tyrer–Cuzick model, and it significantly improved 3–5-year prediction when incorporated into established BC risk models. The MRS was not significantly associated with conventional risk factors or existing risk-assessment scores, supporting its value as a complementary rather than redundant predictor. Notably, it predicted BC risk equally well in individuals with dense and non-dense breasts. Finally, our 22-CpG signature mapped to immune, transcriptional, metabolic, and cell-cycle pathways.

DNA methylation is a stable, cell-type-specific regulatory mark that can both reflect and influence carcinogenesis.^47^ Increased DNA methylation at promoters tends to silence tumour-suppressor genes, whereas decreased DNA methylation at promoters/enhancers of oncogenes can activate their expression. We observed that most BC-associated sites were hypomethylated, in line with previous studies,^48^^,^^49^ which may contribute to genomic destabilisation in immune cells.^50^ Because methylation patterns in blood and breast can overlap,^51^ the observed markers could indicate early defects in genome stability in breast cells or, alternatively, changes in immune cells and/or an altered immune response.^39^^,^^40^^,^^52^ The latter interpretation is supported by previous studies^5^^,^^53^ and by our observation that CpG sites near immune-regulatory genes were successfully replicated in EPIC-Italy. Furthermore, we carefully accounted for (predicted) immune-cell composition in our analyses, suggesting that the MRS captures immune processes beyond leucocyte-proportion differences.^53^ The lack of strong association between the MRS and tumour characteristics suggests that the score may not primarily capture tumour aggressiveness, but rather host-related systemic alterations associated with BC risk or early tumour development. This interpretation is consistent with the enrichment of hypomethylated CpGs near immune-relevant genes and with the relative stability of the MRS across the pre-diagnostic window. However, our functional interpretation is based primarily on genomic annotation and prior literature rather than functional validation; it should therefore be considered hypothesis-generating rather than mechanistic.

Analysing the association between the MRS and BC risk in EPIC-Italy allowed us to further hypothesise about the timeframe during which methylation changes act upon BC risk. The MRS was associated with BC risk (and thus informative for BC risk prediction) only within 0–5 years before diagnosis. This was further exemplified by the results obtained for repeated measurements from the same individual in our study, which showed a stable increase in risk related to methylation changes across the entire 5-year interval. Thus, the risk increase associated with methylation changes in blood appears to be neither acute (i.e., very close to diagnosis) nor chronic (more than 7 years before diagnosis). Importantly, this 5-year interval also provides a practical window with at least two mammography visits to identify individuals in screening programmes who are at higher risk for BC.

When comparing our findings with a previously published methylation-based BC risk score,^8^ no direct overlap was observed between the candidate CpGs from both studies. However, this lack of overlap may reflect several methodological and design differences. The prior BC risk score was developed using Illumina 450 K methylation data and elastic-net selection from a predefined candidate set of CpGs and DNA methylation-based estimators, whereas our MRS was derived using a de novo epigenome-wide discovery approach on the EPIC/850 K array within a population-based mammography screening cohort. In addition, our study focused on a shorter pre-diagnostic interval more directly relevant to near-term BC risk stratification in the screening setting. Notably, both studies identified a predominance of hypomethylated CpGs among the selected markers, suggesting possible convergence at the level of broader biological processes despite differences in the individual CpGs selected.

Several BC risk-prediction models already guide decisions about intensified surveillance or supplemental imaging,^4^^,^^29^ and they increasingly integrate epidemiologic factors, mammographic density, and the 313-SNP PRS (e.g., BOADICEA/CanRisk and Tyrer–Cuzick).^30^^,^^54^, ^55^, ^56^ In our training/test split, the short-term discriminatory power of the MRS (0.5–3 years) was similar or slightly superior to Tyrer–Cuzick and the 313-SNP PRS. As expected, our AI-based mammography model, which provides strong near-term discrimination,^12^ outperformed all other tools. Importantly, adding the MRS produced statistically significant AUC gains for 3–5-year prediction across all established models, highlighting its potential to extend risk stratification beyond traditional inputs. For comparison, other blood-based biomarker classes have not, to date, produced gains comparable to the MRS when added to established risk models. In large prospective affinity-proteomics analyses, neither single proteins nor multi-protein panels remained associated with short-term overall BC risk, and model discrimination was not improved.^31^ Moreover, current circulating tumour DNA assays are primarily designed for detecting prevalent disease rather than forecasting future risk, with no prospective demonstration of improved risk-model performance.^57^ Although the resulting AUCs remain moderate, this is not unexpected in a pre-diagnostic screening setting, where no single blood-based or imaging-based marker is likely to achieve near-perfect discrimination. Rather than serving as a stand-alone prediction tool, the main value of the MRS lies in providing complementary molecular information that improves short-to mid-term risk discrimination when added to existing models, particularly in independent validation analyses.

Strengths of this study include its prospective, population-based design nested within a mammography screening cohort, the use of pre-diagnostic whole-blood samples collected within a screening-relevant time window, and the availability of repeated samples to evaluate temporal stability. The regional discovery-validation design and external replication further strengthened the assessment of reproducibility. In addition, the use of rigorous methylation QC, including correction for BeadChip row effects, reduced the potential influence of technical variation. Finally, comparison with established imaging-, clinical-, and genetic-based risk models allowed us to assess whether blood DNA methylation provides complementary predictive information.

Nevertheless, several limitations/considerations warrant further attention. First, longer follow-up will help refine the temporal window over which the MRS is informative and confirm whether risk discrimination declines beyond 5 years before diagnosis. Repeated sampling in controls would also clarify the stability of MRS values in cancer-free individuals. Second, although the limited sample size means overfitting cannot be entirely ruled out, our pragmatic selection reduced the panel to 22 candidates, and independent replication in EPIC-Italy confirmed 10 of the 13 CpGs available on the 450 K array, supporting the view that this 22-candidate panel merits further validation. Last, prospective trials should evaluate the feasibility, cost-effectiveness, and acceptability of MRS-based screening strategies within mammographic screening cohorts across diverse populations. In addition, the score should also be evaluated in individuals currently not included in screening programmes (i.e., due to being too young to participate) with high (genetic) risk, as changes in the methylation profile could be indicative of imminent BC and thus improve early detection in these individuals as well.

In conclusion, our study identified a DNA methylation signature as a potential early blood-based biomarker of BC risk. Rather than serving as a stand-alone prediction tool, the main value of the MRS may lie in providing complementary short-to mid-term risk information alongside imaging-, clinical-, and genetic-based models.^13^ In settings where blood is already collected for genotyping or ancillary testing, the same specimen could also be used for methylation profiling, offering a practical route to integrate systemic epigenetic signals into BC risk stratification. Larger cohorts, calibration, and decision-curve analyses will be needed to determine clinical utility and net benefit.

## Contributors

Conceptualisation: JR, FG, KC.

Methodology: JR, FG, KC.

Investigation: JR, FG, DK, KC.

Visualisation: JR, FG.

Resources: SH, PH, KC.

Funding acquisition: FG, SH, KC.

Project administration: JR, FG, KC.

Supervision: KH, KC.

Writing – original draft: JR, FG, KC.

Writing – review & editing: JR, FG, DK, SH, KH, ME, YZ, KM, MG, PH, KC.

All authors read and approved the final version of the manuscript. JR and FG had full access to raw data and were responsible for the decision to submit for publication.

## Data sharing statement

Access to KARMA phenotypes, genotype data, DNA methylation data, derived risk scores, and related study data can be requested through the KARMA data access process at https://karmastudy.org/data-access/. Requests are reviewed according to KARMA governance procedures and require an approved research proposal, relevant ethical approvals, and a data transfer or data use agreement, where applicable. Analysis code is available from the corresponding author upon reasonable request.

## Declaration of interests

The authors declare no potential conflicts of interest.
