# Supplementary material for: Blood DNA methylation and breast cancer risk: a prospective nested case–control study
Source: eBioMedicine. 2026 Jun 25;129:106352. doi: 10.1016/j.ebiom.2026.106352 (PMC13320414; doi:10.1016/j.ebiom.2026.106352)
Supplement: Supplementary Figs. S1–S7 and Tables S1 and S2 [file mmc1.pdf]

<u>Supplementary material</u>

Supplementary Table S1. Baseline characteristics of all index breast cancer cases and controls.

Supplementary Figure S1. Distribution of breast cancer cases by time from blood draw to diagnosis.

Supplementary Figure S2. Flowchart of the DNA methylation quality-control pipeline.

Supplementary Figure S3. Principal component analysis scatter plots of DNA methylation data.

Supplementary Figure S4. Manhattan plot of epigenome-wide association *P* values across autosomal chromosomes.

Supplementary Figure S5. Correlation matrix of the 22 validated CpG sites associated with breast cancer.

Supplementary Figure S6. Association of breast cancer risk factors with MRS.

Supplementary Figure S7. External replication in EPIC-Italy.

Supplementary Table S2. Functional annotation of 22 validated CpG sites associated with breast cancer risk.

Supplementary Table S1. Baseline characteristics of all index breast cancer cases and controls, stratified by recruitment centre.

Baseline Characteristics	All index blood-draws				Stockholm subset				Skåne subset			
	Cases (N=377)	Controls (N=378)	*P*	Missing (%)	Cases (N=196)	Controls (N=191)	*P*	Missing (%)	Cases (N=181)	Controls (N=187)	*P*	Missing (%)
Recruitment Centre, n (%)												
Stockholm	196 (52)	191 (50.5)	0.74	0								
Skåne	181 (48)	187 (49.5)										
Age at blood draw, mean SD)	58.8 (9.1)	58.8 (9.7)	0.96	0	58.7 (8.9)	59.2 (9.5)	0.563	0	58.9 (9.3)	58.4 (9.9)	0.62	0
BMI, mean (SD)	25.5 (4.3)	25.3 (4.3)	0.41	2.3	25.4 (4.4)	25 (4.3)	0.380	2.6	25.7 (4.3)	25.6 (4.3)	0.777	1.9
Smoking, n (%)												
Never	147 (39)	168 (44.4)	0.11	4.8	70 (35.7)	80 (41.9)	0.207	4.6	77 (42.5)	88 (47.1)	0.603	4.9
Previously	162 (43)	150 (39.7)			88 (44.9)	80 (41.9)			74 (40.9)	70 (37.4)		
Currently	44 (11.7)	48 (12.7)			25 (12.8)	26 (13.6)			19 (10.5)	22 (11.8)		
Missing	24 (6.4)	12 (3.2)			13 (6.6)	5 (2.6)			11 (6.1)	7 (3.7)		
Education level, n (%)												
Elementary	44 (11.7)	53 (14)	0.35	6.6	22 (11.2)	21 (11)	0.736	6.2	22 (12.2)	32 (17.1)	0.367	7.1
Intermediate	128 (34)	121 (32)			65 (33.2)	58 (30.4)			3 (34.8)	63 (33.7)		
University	175 (46.4)	184 (48.7)			95 (48.5)	102 (53.4)			80 (44.2)	82 (43.9)		
Missing	30 (8)	20 (5.3)			14 (7.1)	10 (5.2)			16 (8.8)	10 (5.3)		
Mammographic density, mean (SD)	23.6 (19.4)	20.5 (18.6)	**0.028**	0.7	23.3 (20)	21.8 (19.9)	0.453	0.3	23.9 (18.8)	19.2 (17.2)	**0.014**	1.1
Parity, mean (SD)	1.88 (1)	2.03 (1)	0.055	6.9	1.78 (1)	1.85 (1)	0.531	8.3	1.98 (1)	2.2 (1)	**0.036**	5.4
Menarche age, mean (SD)	13.1 (1.4)	13.2 (1.4)	0.302	7.5	12.9 (1.4)	13.1 (1.6)	0.346	8.0	13.2 (1.4)	13.3 (1.2)	0.665	7.1
Age at first birth, mean (SD)	27.3 (5.6)	26.2 (5.1)	**0.01**	0	27.6 (5.8)	26.8 (5.2)	0.237	0	27 (5.4)	25.6 (4.9)	**0.012**	0
Alcohol gram/week, mean (SD)	55.4 (59)	46.2 (59.9)	**0.041**	7.3	60.4 (67.9)	48.1 (63.5)	0.081	8.5	50.2 (47.7)	44.2 (56.2)	0.286	6
Menopause status, n (%)												
Premenopausal	130 (34.5)	123 (32.5)	0.625	0	65 (33.2)	59 (30.9)	0.711	0	65 (35.9)	64 (34.2)	0.818	0
Postmenopausal	247 (65.5)	255 (67.5)			131 (66.8)	132 (69.1)			116 (64.1)	123 (65.8)		
Family history of breast cancer, n (%)												

No	287 (76.1)	312 (82.5)	**<0.001**	4.5	148 (75.5)	159 (83.2)	**0.001**	4.7	139 (76.8)	153 (81.8)	**0.002**	4.3
Yes	82 (21.8)	40 (10.6)			43 (21.9)	19 (9.9)			39 (21.5)	21 (11.2)		
Missing	8 (2.1)	26 (6.9)			5 (2.6)	13 (6.8)			3 (1.7)	13 (7.0)		

Data are presented as mean (standard deviation, SD) for continuous variables and as counts (%) for categorical variables. Differences between breast cancer cases and controls were evaluated using t tests for continuous variables and chi-square tests for categorical variables. Age at first birth is summarized only among parous women. Variables with statistically significant differences ($P < .05$) between groups are shown in boldface. Missing values are shown as percentages for each variable.

Supplementary Figure S1. Distribution of breast cancer cases in the entire study cohort (A), and separately for the Stockholm (B) and Skåne (C) regions, by time from blood draw to diagnosis.

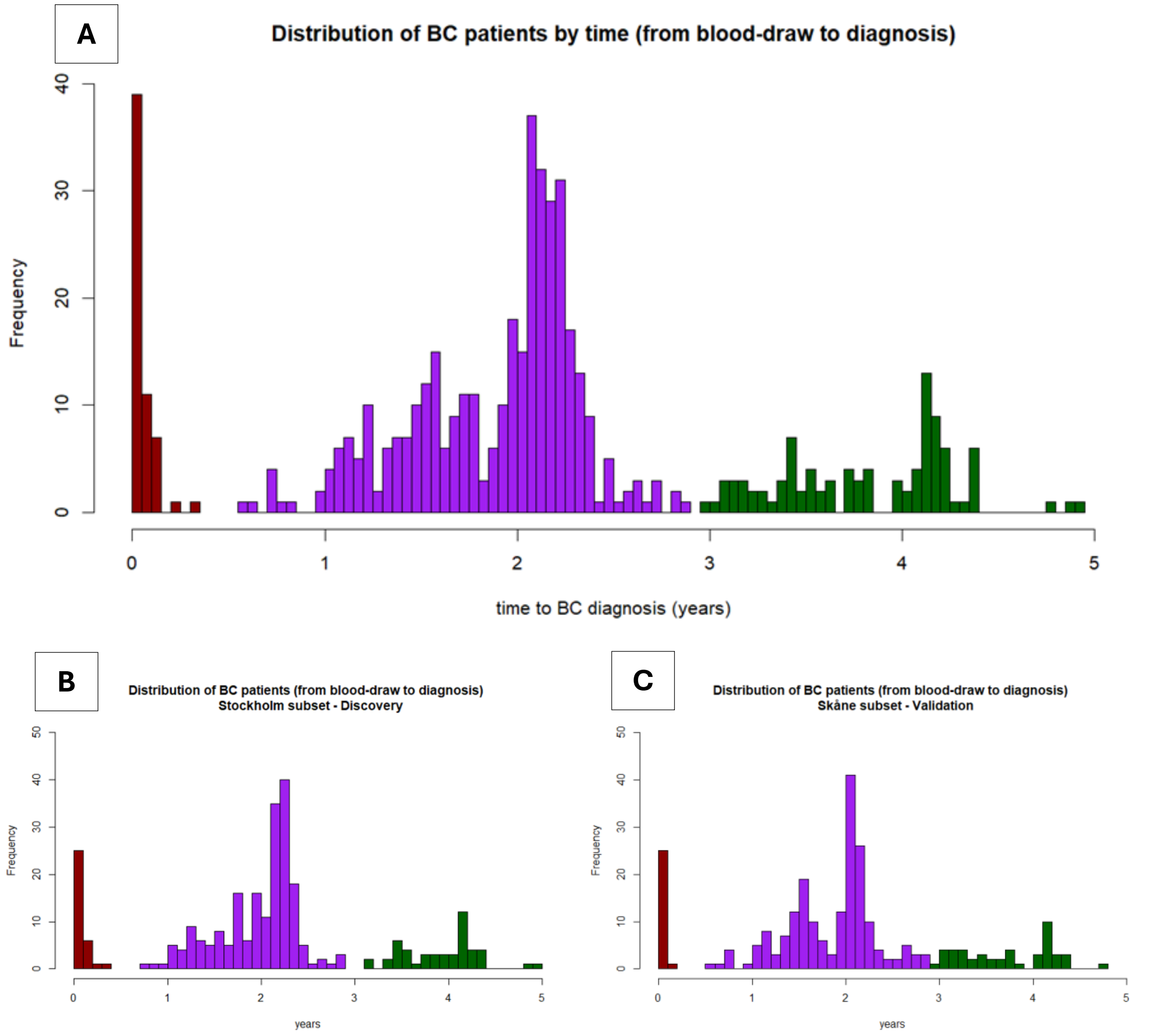

Purple bars consist of all unique patients with BC from the combined Stockholm and Skåne regions (**Panel A** N=377), from Stockholm set only (**Panel B** N=196) and Skåne set (**Panel C** N=181), who were diagnosed with BC 6 months to 3 years after the blood draw. As the blood draw was taken during the last screening visit that was considered negative for BC, we refer to these samples as the index blood draws in this study. Green (**Panel A** N=96; **Panel B** N=49; **Panel C** N=47) and red (**Panel A** N=59; **Panel B** N=33; **Panel C** N=26) areas consist of secondary blood draws (subset of the purple group) obtained before and after the index blood draw, respectively.

Abbreviation: *BC,* breast cancer.

Supplementary Figure S2. Flowchart of the DNA methylation quality-control pipeline.

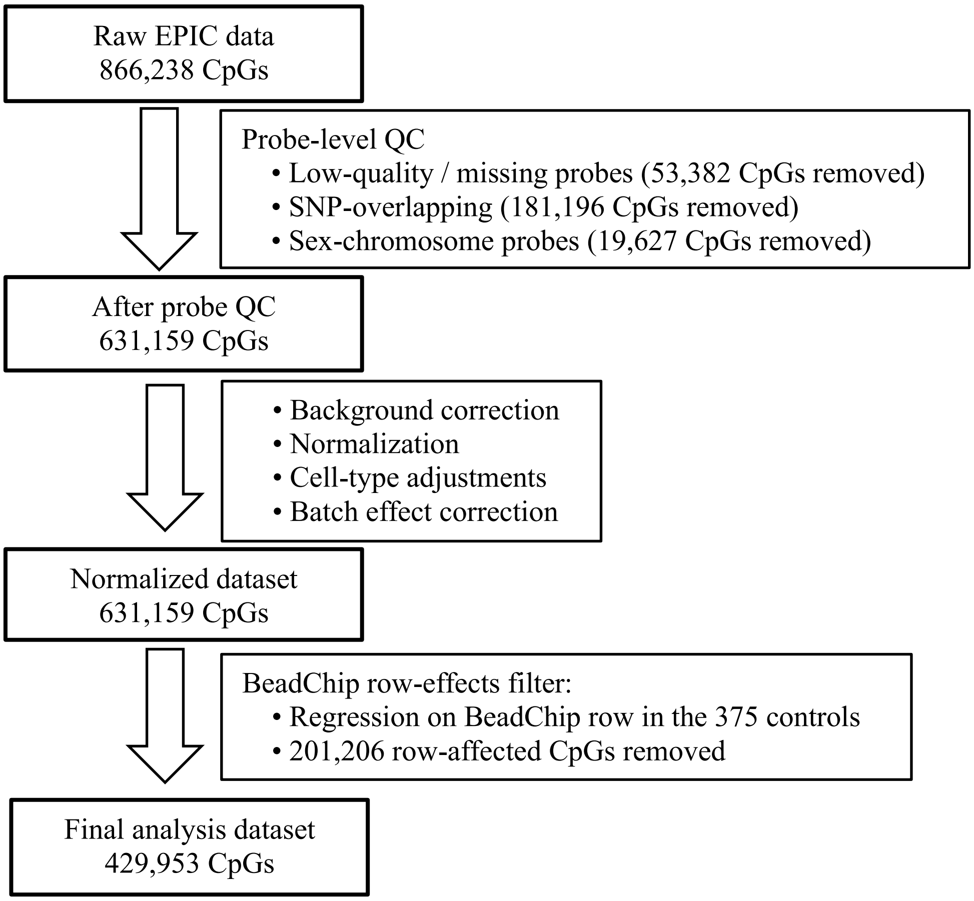

The Flowchart illustrates the multi-step quality-control (QC) pipeline that the methylation data went through.

Supplementary Figure S3. Principal component analysis scatter plots of DNA methylation data.

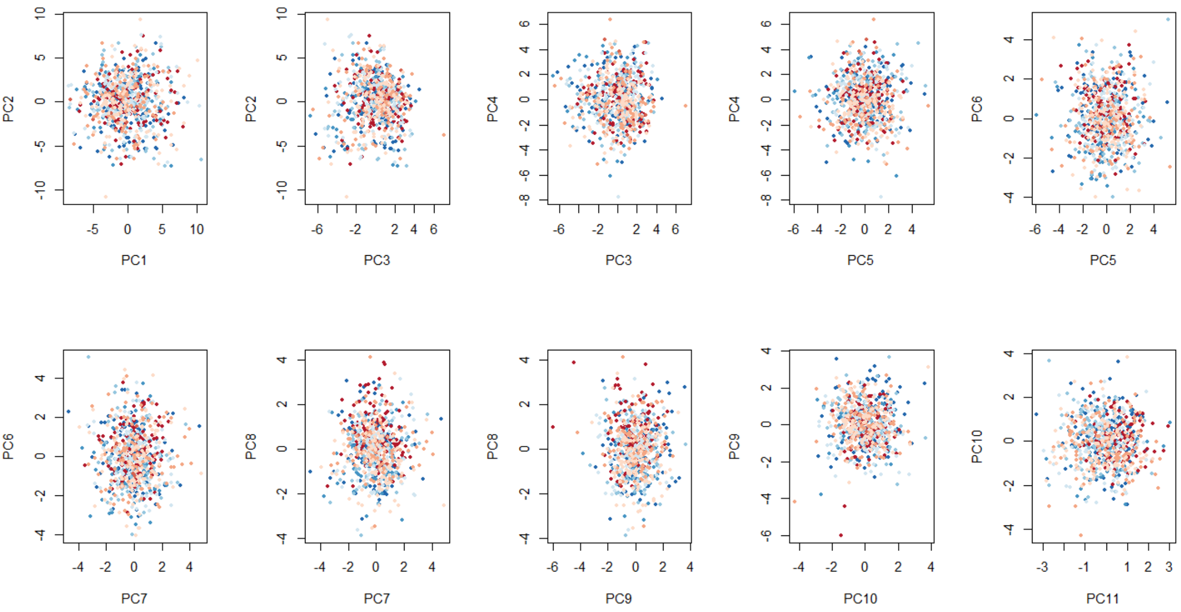

Pairwise scatter plots of principal components (PCs) derived from adjusted DNA methylation β values in all index blood draw cases and controls. Each point represents an individual sample, coloured according to physical array position. Axes correspond to standardized values of specific PCs (e.g., PC1 vs. PC2, PC3 vs. PC4), capturing major axes of variation in the methylation data.

Supplementary Figure S4. Manhattan plot of epigenome-wide association P values across autosomal chromosomes.

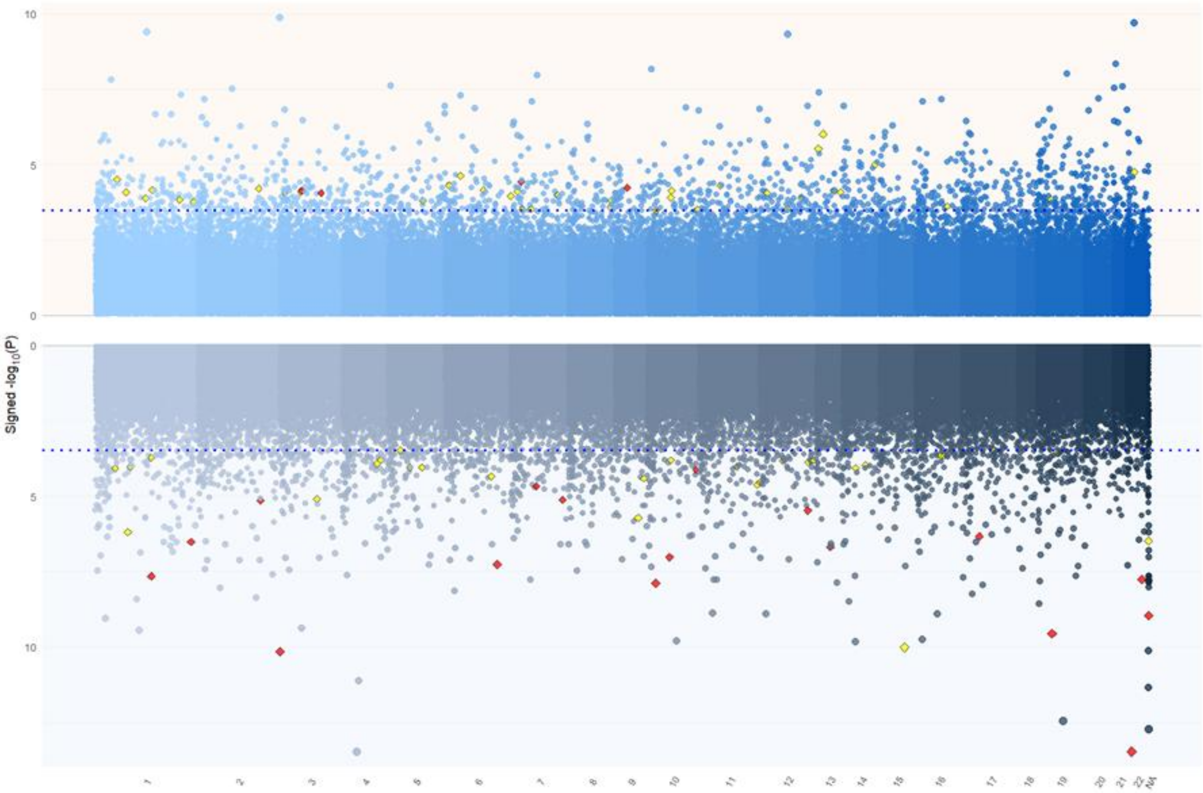

Manhattan plot of epigenome-wide association *P* values for CpG sites across autosomal chromosomes. The vertical axis represents signed -log10(*P*) values from the discovery analysis in the Stockholm set, with the sign indicating the direction of the regression coefficient. Yellow diamonds highlight CpGs significant in Stockholm (FDR < 0.05, |β| > 0.015). Red diamonds indicate CpGs significant in both Stockholm (FDR < 0.05, |β| > 0.015) and Skåne (*P* < 0.05) with consistent direction of effect. Dashed lines indicate the *P* value threshold corresponding to FDR < 0.05, as determined in the Stockholm analysis.

Supplementary Figure S5. Correlation matrix of the 22 validated CpG sites associated with breast cancer.

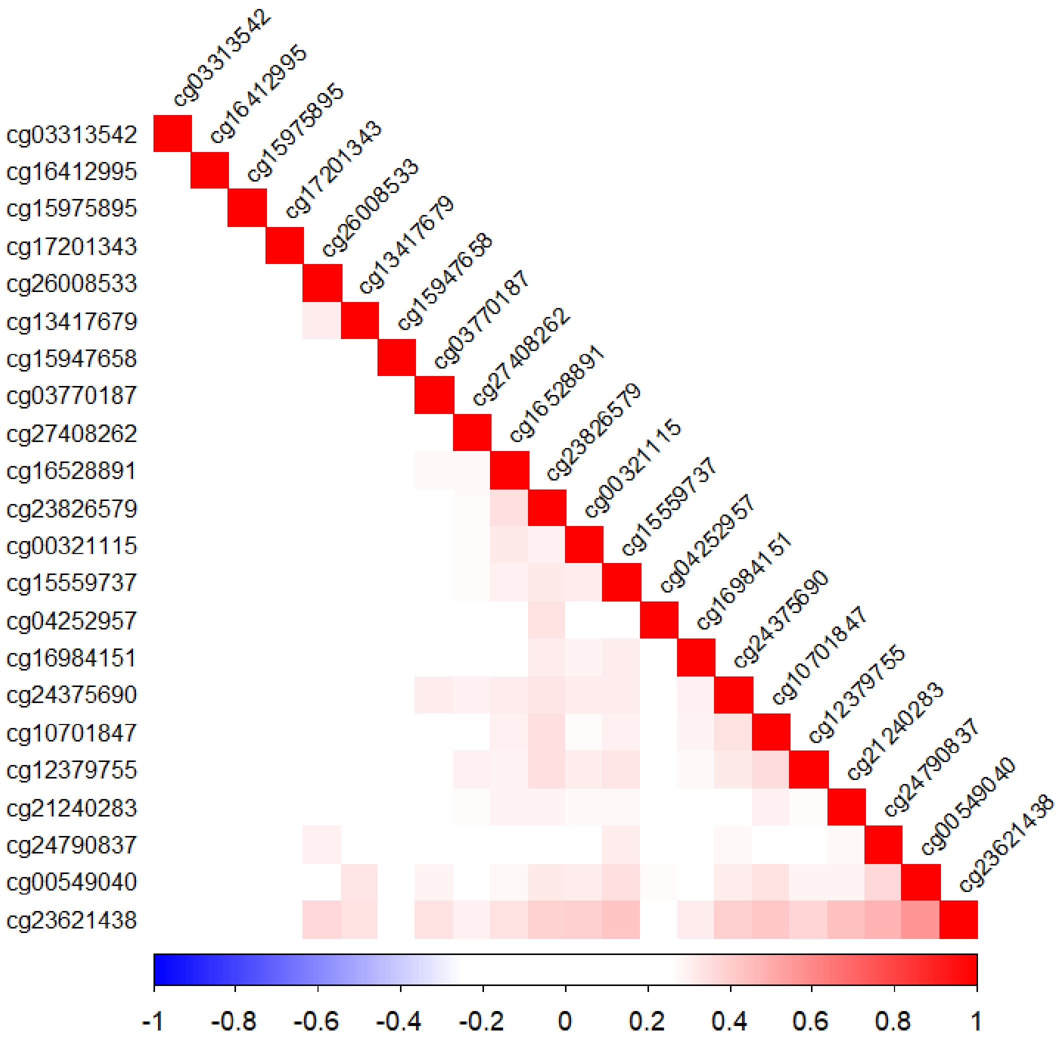

The heatmap displays pairwise Pearson correlation coefficients between the 22 CpG sites that were significantly associated with breast cancer in the Stockholm set and showed replication in the Skåne set (FDR < 0.05 and consistent direction of effect). Colours represent the strength and direction of the correlation, with blue indicating negative correlation and red indicating positive correlation. Correlations were calculated using β values across all participants included in the analysis.

Supplementary Figure S6. Association of breast cancer risk factors with MRS.

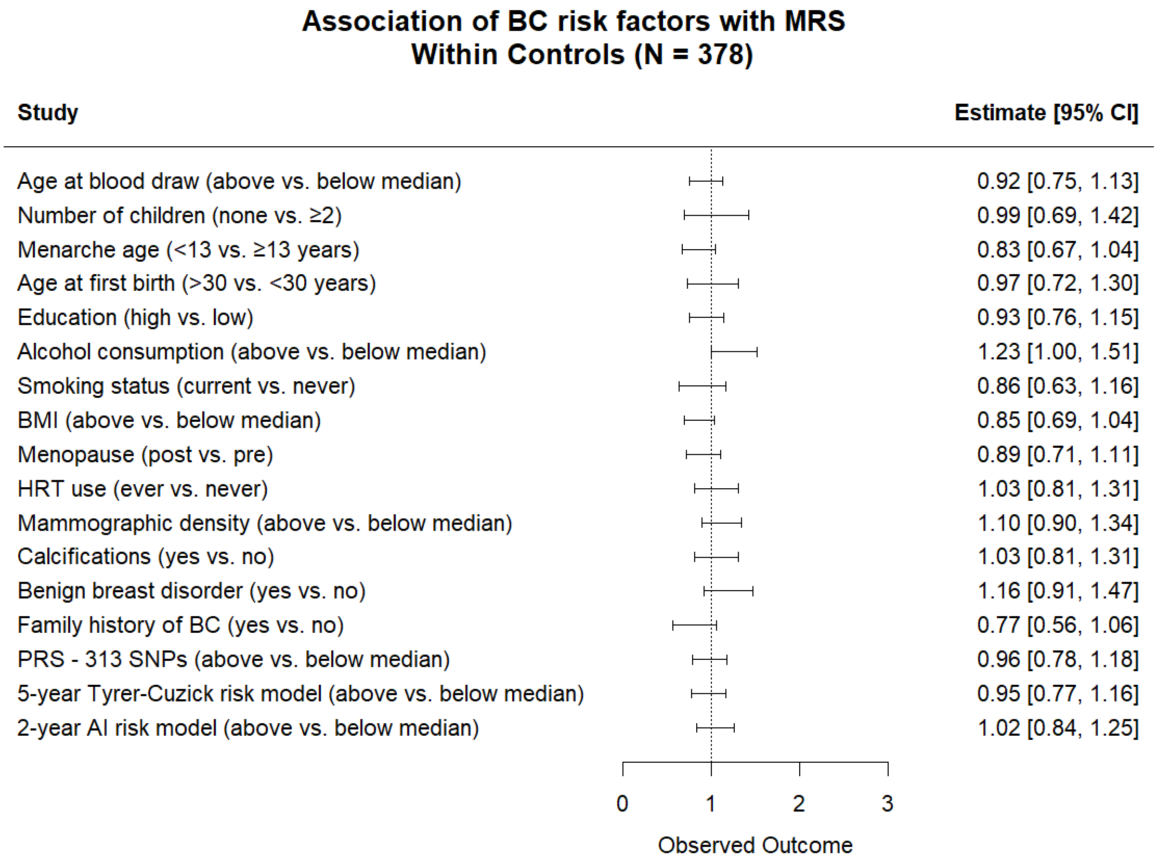

Forest plot displaying ORs and 95% CIs for the association between the scaled MRS and established BC risk factors among control participants (N=378). Each estimate is derived from a separate logistic regression model with the risk factor as the outcome (e.g., age above vs below the median, high vs low BMI) and the scaled MRS as the predictor. Additional risk prediction scores (PRS - 313SNPs, 5-year Tyrer-Cuzick, and 2-year AI risk models) are also evaluated for their association with MRS. The vertical dashed line indicates the null value (OR = 1).

Abbreviations: *BC,* breast cancer; *CI,* confidence interval; *PRS,* polygenic risk score; *MRS,* methylation risk score; *OR,* odds ratio.

Supplementary Figure S7. External replication in EPIC-Italy.

A

CpG ID	Gene symbol (UCSC RefGene)	EPIC-Italy			Pooled meta-analysis		
		Δβ	SE	*P*	Δβ	SE	*P*
cg12379755	—	-0.0389	0.0053	1.00E-12	-0.01280	0.00230	2.77E-08
cg24375690	*BSPH1*	-0.0206	0.0040	3.69E-07	-0.01509	0.00250	2.71E-09
cg16528891	—	-0.0222	0.0045	1.03E-06	-0.01440	0.00230	4.19E-10
cg23826579	*MICA*	-0.0213	0.0046	5.10E-06	-0.01422	0.00200	5.59E-12
cg15559737	*CRYBA4*	-0.0169	0.0038	1.23E-05	-0.01238	0.00180	1.13E-11
cg21240283	*FAM3C*	-0.0179	0.0043	3.73E-05	-0.01477	0.00280	1.08E-07
cg00321115	*FLT1*	-0.0164	0.0040	3.96E-05	-0.01152	0.00220	1.82E-07
cg27408262	*CTBP2*	-0.0176	0.0043	4.39E-05	-0.01277	0.00200	5.77E-10
cg10701847	*FAM113B*	-0.0167	0.0043	9.23E-05	-0.01140	0.00230	1.22E-06
cg03770187	*OR14A16*	-0.0137	0.0038	3.19E-04	-0.01407	0.00240	8.94E-09
cg04252957	*DHCR7*	-0.0087	0.0045	5.50E-02	-0.01178	0.00210	2.46E-08
cg00549040	—	-0.0028	0.0051	5.82E-01	-0.01437	0.00180	1.67E-14
cg03313542	*TMEM209*	-0.0038	0.0086	6.55E-01	0.02120	0.00470	9.25E-06

B

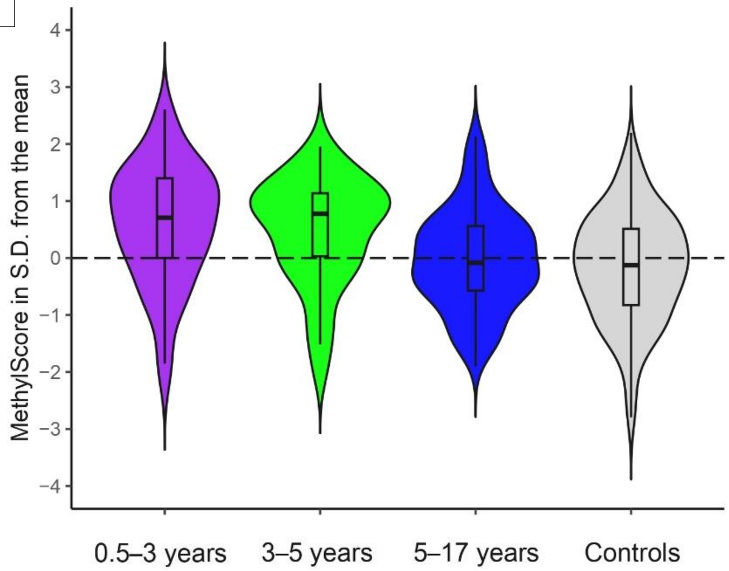

C

MRS SwedenTraining/Test split vs. MRS on EPIC Italy			
Index blood draw vs. EPIC Italy *0.5–3 years to BC diagnosis*	N cases	N controls	AUC (95% CI)
MRS Sweden (Training/Test split)	181	187	0.66 (0.61–0.72)
MRS Sweden on EPIC Italy	80	340	0.71 (0.64–0.78)
Secundary blood draw vs. EPIC Italy *3–5 years to BC diagnosis*	N cases	N controls	AUC (95% CI)
MRS Sweden (Training/Test split)	47	187	0.71 (0.62–0.79)
MRS Sweden on EPIC Italy	34	340	0.7 (0.6–0.8)
Secundary blood draw vs. EPIC Italy *5–17 years to BC diagnosis*	N cases	N controls	AUC (95% CI)
MRS Sweden (Training/Test split)	-	-	-
MRS Sweden on EPIC Italy	114	340	0.53 (0.47–0.59)

Panel A shows all significant CpG sites and corresponding RefSeq gene symbols that were present on the Illumina 450K array in the EPIC-Italy study (https://www.ncbi.nlm.nih.gov/geo/query/acc.cgi?acc=GSE51032). Association statistics are presented separately for the EPIC-Italy replication and for the pooled Swedish meta-analysis. The last three rows (highlighted in red) indicate CpG sites that did not replicate. **Panel B** shows violin plots of the MRS among patients with BC and controls in the EPIC-Italy cohort, stratified by time from blood draw to diagnosis. Each violin plot shows the distribution of MRS values (expressed in SD units), with an overlaid boxplot indicating the interquartile range and median. Time windows were defined as 0.5–3, 3–5, and 5–17 years before BC diagnosis. **Panel C** shows AUC values (95% CI) comparing the Swedish MRS (developed in the Stockholm set and evaluated in the Skåne set) with the MRS computed in EPIC-Italy using the 13 available CpGs. EPIC-Italy models used pooled meta-analysis weights and logistic regression. Models were adjusted for age and array row. NOTE: Only 5 patients with BC in EPIC-Italy had blood drawn less than 6 months before diagnosis; therefore, this time window was not evaluated in the replication analysis.

Abbreviations: *AUC,* area under the curve; *BC,* breast cancer; *CI,* confidence interval; *MRS,* methylation risk score; *SD,* standard deviation.

Supplementary Table S2. Functional annotation of 22 validated CpG sites associated with breast cancer risk.

CpG ID	CHR	Genomic position	*P* Pooled	Effect (Δβ) Pooled	Gene symbol	Region group
cg23621438	22	25850271	5.50E-19	-0.0135	*MIR6817; CRYBB2P1*	TSS1500; Body
cg00549040	2	218048915	1.67E-14	-0.0144		
cg23826579	6	31373932	5.59E-12	-0.0142	*MICA*	Body
cg15559737	22	27016806	1.13E-11	-0.0124	*CRYBA4*	TSS1500
cg16528891	1	36169528	4.19E-10	-0.0144		
cg27408262	10	126706831	5.77E-10	-0.0128	*CTBP2*	Body
cg24375690	19	48495961	2.71E-09	-0.0151	*BSPH1*	TSS1500
cg03770187	1	247979766	8.94E-09	-0.0141	*OR14A16*	TSS1500
cg16984151	10	35540547	1.91E-08	-0.0151	*CCNY*	5'UTR
cg04252957	11	71147213	2.46E-08	-0.0118	*DHCR7*	Body
cg12379755	17	62343790	2.77E-08	-0.0128		
cg13417679	10	31284225	4.46E-08	-0.0162	*ZNF438*	Body; 5'UTR
cg21240283	7	121032965	1.08E-07	-0.0148	*FAM3C*	5'UTR
cg00321115	13	28942568	1.82E-07	-0.0115	*FLT1*	Body; 3'UTR
cg15975895	3	178668607	1.83E-07	0.0161		
cg17201343	9	109655439	2.43E-07	0.0146	*ZNF462; MIR548Q*	5'UTR; Body
cg15947658	22	43584066	3.77E-07	-0.0132	*TTLL12*	TSS1500
cg10701847	12	47626844	1.22E-06	-0.0114	*FAM113B*	5'UTR
cg24790837	7	138838713	1.29E-06	-0.0119	*TTC26*	Body
cg16412995	3	196249660	3.71E-06	0.0119		
cg26008533	2	32389741	6.52E-06	-0.0143	*SLC30A6*	TSS1500
cg03313542	7	129845597	9.25E-06	0.0212	*TMEM209*	TSS1500

Summary of gene annotations for the 22 validated CpG sites. Columns include CpG identifier, chromosome (CHR), genomic position (hg19), effect estimate (Δβ) and *P* value from the pooled meta-analysis, the RefSeq gene symbol, and region group (UCSC "RefGene" track).
